# Restaurant Inspection Letter Grades and *Salmonella* Infections, New York, New York, USA 

**DOI:** 10.3201/eid2412.180544

**Published:** 2018-12

**Authors:** Melanie J. Firestone, Craig W. Hedberg

**Affiliations:** University of Minnesota School of Public Health, Minneapolis, Minnesota, USA; Minnesota Integrated Food Safety Center of Excellence, Minneapolis

**Keywords:** *Salmonella*, restaurants, food inspection, restaurants, bacteria, food safety, enteric infections, New York City, United States

## Abstract

Rates of *Salmonella* infection in the United States have not changed over the past 20 years. Restaurants are frequent settings for *Salmonella* outbreaks and sporadic infections. Few studies have examined the effect of posting letter grades for restaurant inspections on the incidence of foodborne illness. We compared *Salmonella* infection rates in New York, New York, USA (NYC), with those in the rest of New York state before and after implementation of a letter grade system for restaurant inspections in NYC. We calculated a segmented regression model for interrupted time series data. After implementation of letter grading, the rate of *Salmonella* infections decreased 5.3% per year in NYC versus the rest of New York state during 2011–2015, compared with the period before implementation, 2006–2010. Posting restaurant inspection results as letter grades at the point of service was associated with a decline in *Salmonella* infections in NYC and warrants consideration for broader use.

Each year, an estimated 48 million persons get sick, 128,000 are hospitalized, and 3,000 die from foodborne illness in the United States (*1*). Consumption of food prepared away from home, such as at restaurants and for takeout, has continuously increased, accounting for 50% of food expenditures in 2014, up from 33% in 1970 (*2*). Restaurants are frequent settings for transmission of foodborne illness; in 2015, 60% of foodborne illness outbreaks were associated with restaurants (*3*). There is also evidence that commercial food service establishments, such as restaurants, play a role in sporadic (nonoutbreak) cases of foodborne illness (*4*). Given our dependence on food prepared away from home, reducing the risk for foodborne illness from commercial food service establishments is of critical importance.

Food service establishments play a role in the epidemiology of *Salmonella* infections; *Salmonella* may contaminate a wide range of raw ingredients, infect food workers, survive on contaminated surfaces, and grow in improperly held food items. Nontyphoidal *Salmonella* infections are the second most common foodborne illness and the leading cause of foodborne illness hospitalizations and death (*1*). Reducing *Salmonella* infections is a Healthy People 2020 objective (*5*), yet rates have not substantially changed over the last 20 years (*6*). Inspection of food service establishments to protect food safety is a core function of state and local health departments; the inspections help to identify risk factors for foodborne illness, such as those associated with *Salmonella* transmission, and to correct them, thus protecting consumers and industry.

New York does not have a statewide policy for reporting restaurant inspection results. In 2005, the Department of Health and Mental Hygiene (DOHMH) in New York, NY, USA (NYC), began using a point-scoring system for food service establishment inspections to weight violations to reflect risk factors for foodborne illness. In 2010, the NYC DOHMH implemented a letter grade program that converted these scores into categorical rankings of A, B, or C, or grade pending, in an effort to improve restaurant food safety, increase transparency of inspection information, and reduce the risk for foodborne illness transmission in restaurants (*7*). The DOHMH required establishments to post a sign with the letter grade in its window so that consumers could see it before entering. Public disclosure programs like this one seek to provide information to consumers when and where they need it so they can make informed decisions about potential risks (*8*). These consumer decisions encourage restaurant operators to improve and maintain sanitary standards, thus improving sanitary conditions in restaurants. The letter grade program has already been shown to lead to improvements in sanitary conditions in NYC: 35% more restaurants earned A grades in the 3 years after grading, compared with the 3 years before (*9*).

The value of posting restaurant inspection ratings at the point of service has been the subject of considerable debate. Few studies have looked at the impact of posting policies on the incidence of foodborne illness. Two studies of a letter grade program in Los Angeles County, California, USA, showed a reduction in foodborne-illness hospitalizations (*10**,**11*). In NYC, a preliminary analysis of letter grading at 18 months suggested a decline in *Salmonella* infections. The goal of our study was to compare the incidence of *Salmonella* infections in NYC with incidence in the rest of the state before and after the implementation of posting letter grade placards at the point of service.

## Methods

### Data

*Salmonella* infections are nationally notifiable (*12*). We obtained yearly laboratory-confirmed case counts from 1994–2015 from the NYC DOHMH and the New York State Department of Health. Cases are reported by year of diagnosis and county of residence.

### Statistical Analyses

To account for population changes, we calculated annual rates using intercensal population estimates for 1994–2015 from the US Census Bureau. We calculated the percent change from year to year. We compared mean rates of *Salmonella* infection before and after implementation of a point scoring system in 2005 and after implementation of grade cards in 2010 using *t*-tests for NYC and the rest of the state (NYS). We considered the year of implementation to be a part of the before period in both analyses because the policies were not implemented on January 1 of each year.

We used segmented regression to determine the trend before implementing the policy in *Salmonella* infections, an immediate change at the time of policy implementation, and the long-term trend after policy implementation (*13*). We hypothesized that the long-term trend would decline after policy implementation. We expected a delayed effect because restaurants are not all on the same inspection cycle and because underlying improvements in sanitation driving the decline are not likely to be immediate. Because there were indications of overdispersion and heteroskedasticity, we used a negative binomial regression model with robust SEs to quantify the effect of letter grade placards on *Salmonella* infections. We used an offset term to account for population changes across the period. Examination of autocorrelation and partial autocorrelation functions confirmed that the outcome was not autocorrelated. We calculated incidence rate ratios (IRR) comparing *Salmonella* infections in NYC to NYS before and after the implementation of the point scoring system and posting of letter grade placards.

Key variables included year, coded as a continuous variable starting with 1994 = 1; variables representing the 2 policy periods (prepolicy = 0, postpolicy = 1); 2 variables representing the trends after policy implementation, coded as 0 before the policy and a continuous numerical function after the policy was implemented; and a variable (region) representing NYC versus NYS. The model also included 5 interaction terms: region by year, to control for regional secular trends; region by policy for each policy (before and after letter grade was implemented), to account for the mean level change in NYC after the new policy took effect; and region by trend before and after policy implementation, to determine if the average *Salmonella* infections in NYC changed after policy implementation versus NYS (*14*). We also conducted a subanalysis using data from 2000–2015 to compare NYC to surrounding counties (NYC metropolitan area) and NYS. We analyzed data using Stata version 14.2 (StataCorp LLC, College Station, TX, USA) and determined statistical significance as p<0.05.

## Results

The annual rate of *Salmonella* infections decreased in both NYC and NYS during 1994–2015 (Table 1; Figure). The period-to-period percent change after letter grading implementation was a decline of 32.6% in NYC, compared with a decline of 14.1% in NYS (Table 1). Mean *Salmonella* infection rates in NYC between 1994 and 2010 were significantly higher (p<0.01) than in NYS (Table 2). In the period after letter grading was implemented (2011–2015), the mean rate of *Salmonella* infection was no longer significantly different (p = 0.37) in NYC (mean 12.6 cases/100,000 persons; 95% CI 10.9–14.4) compared with NYS (mean 12.0 cases/100,000 persons; 95% CI 11.4–12.6).

**Table 1:** Confirmed *Salmonella* infection case counts, rates, and percent changes for New York, NY, USA, and the rest of the state, 1994–2015*

Year	NYC				NYS		
No. cases	Rate per 100,000 population	Year-to-year change, %	No. cases	Rate per 100,000 population	Year-to-year change, %
1994	1,890	25.0	–		1,977	18.2	–
1995	2,166	28.4	13.7		1,912	17.6	−3.3
1996	1,927	25.0	−11.8		1,940	17.8	1.5
1997	1,772	22.8	−8.9		1,649	15.2	−14.9
1998	1,751	22.3	−2.3		1,680	15.4	1.7
1999	1,508	19.0	−14.8		1,516	13.9	−10.1
2000	1,215	15.2	−20.1		1,293	11.8	−15.1
2001	1,386	17.2	13.5		1,397	12.7	7.7
2002	1,458	18.1	5.0		1,613	14.6	15.0
2003	1,307	16.2	−10.3		1,282	11.5	−20.8
2004	1,273	15.8	−2.3		1,291	11.6	0.5
2005†	1,203	15.0	-5.1		1,427	12.8	10.6
2006	1,272	15.9	6.0		1,423	12.8	−0.2
2007	1,304	16.3	2.3		1,476	13.3	3.7
2008	1,268	15.7	−3.4		1,491	13.4	0.8
2009	1,236	15.2	−3.3		1,370	12.3	−8.4
2010‡	1,304	15.9	4.7		1,448	12.9	5.4
2011	1,125	13.6	−14.7		1,423	12.7	−1.9
2012	1,171	14.0	3.1		1,395	12.4	−2.0
2013	1,124	13.3	−4.8		1,300	11.6	−6.9
2014	987	11.6	−12.8		1,320	11.7	1.6
2015	918	10.7	−7.8		1,314	11.7	0.0
Period-to-period change, 1994–2005 vs. 2006–2010			−22.8				−10.6
Period-to-period change, 2006–2010 vs. 2011–2014			−20.0				−7.1
Period-to-period change, 1994–2010 vs. 2011–2015			−32.6				−14.1

*Percentages were calculated from unrounded values; the values shown may be different when calculated from the rounded values in the table. NYC, New York City; NYS, rest of New York state.  †Year of implementation of point scores for food safety inspections. ‡Year of implementation of letter grades for food safety inspections.

**Figure:**
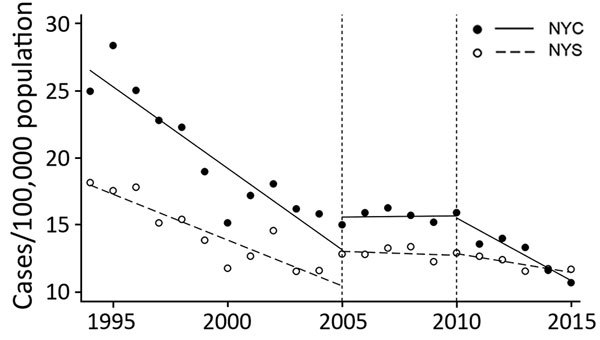
Confirmed *Salmonella* infection cases per 100,000 population in NYC and the rest of the state, 1994–2015. Dashed lines indicate implementation of point scores in 2005 and letter grades in 2010. NYC, New York City; NYS, rest of state.

**Table 2:** Mean *Salmonella* infection rates for New York, NY, USA, and the rest of the state, by policy implementation period, 1994–2015*

Location	1994–2005				2006–2010				2011–2015		
	Mean (SE)	95% CI	p value		Mean (SE)	95% CI	p value		Mean (SE)	95% CI	p value
NYC	20.0 (1.3)	17.1–22.9	Ref		15.8 (0.2)	15.3–16.3	0.06‡		12.6 (0.6)	10.9–14.4	<0.01‡
NYS	14.4 (0.7)	12.9–16.0	Ref		12.9 (0.2)	12.4–13.5	0.21‡		12.0 (0.2)	11.4–12.6	0.01‡
NYC vs. NYS†			<0.01				<0.01				0.37

*NYC, New York City; Ref, referent; NYC, rest of New York state. †Comparison of mean rate between NYC and NYS within time period. ‡Comparison of mean rate within region to the preceding time period.

In NYC and NYS, *Salmonella* infections were decreasing before either policy was implemented in NYC (IRR 0.95; 95% CI 0.94–0.96; p<0.01). The interaction term for trend by region after letter grade implementation was statistically significant (p<0.01), which indicates that *Salmonella* infection rates declined on average in NYC versus NYS in the years after letter grading was implemented, compared with the period after the point scoring system was implemented (Table 3). After letter grading was implemented, the rate of *Salmonella* infections decreased 5.3% per year in NYC versus NYS (IRR 0.95; 95% CI 0.92–0.98; p<0.01).

**Table 3:** Regression for interrupted time series analysis of *Salmonella* infection rates, New York, NY, USA, and the rest of the state, 1994–2015*

Predictor variables	IRR	Robust SE	95% CI	p value
Year	0.95	0.01	0.94–0.96	<0.01
Region	1.50	0.08	1.35–1.68	<0.01
Region × year	0.99	0.01	0.97–1.00	0.16
Point scoring policy implementation	1.20	0.06	1.08–1.32	<0.01
Trend after point scoring implementation	1.04	0.01	1.02–1.07	<0.01
Region × point scoring implementation	0.92	0.07	0.80–1.07	0.29
Region × trend after points	1.02	0.02	0.99–1.05	0.28
Letter grade policy implementation	1.01	0.04	0.93–1.10	0.81
Trend after grading	0.98	0.01	0.96–1.00	0.09
Region × grading	0.99	0.06	0.88–1.11	0.85
Region × trend after grading	0.95	0.02	0.92–0.98	<0.01
Intercept	0.00	0.00	0.00–0.00	<0.01

*IRR, incidence rate ratio.

In a subanalysis of *Salmonella* infections from 2000–2015 comparing number of infections in NYC with that of other counties in the NYC metropolitan area and NYS separately, *Salmonella* infections declined in both NYC and the NYC metropolitan area compared with those in NYS. In contrast to the findings in NYS, in the period after letter grading was implemented, NYC *Salmonella* infections declined 8.8% per year and *Salmonella* infections in the NYC metropolitan area declined 7.5% per year compared with the period between the implementation of a point scoring system and the letter grade program.

## Discussion

Overall, *Salmonella* infections declined in NYC and NYS between 1994 and 2015. Although NYS had declines in *Salmonella* infection rates after 2010, NYC saw declines greater than those in NYS. Inspection processes were largely unchanged in NYC with the implementation of the point scoring system in 2005 (*15*). The letter grade placard program begun in 2010 did not change the point scoring system but rather used the points to create a readily comprehensible ranking system accessible at the point of service. Our analysis supports the hypothesis that having a point scoring system was not associated with declines in *Salmonella* infections but having a simple way to publicly disclose the results of the inspection was.

Although it appears that the implementation of a point scoring system in NYC was associated with a leveling off of declines in *Salmonella* infections, the system was implemented at the end of a hyperendemic period of *Salmonella enterica* serotype Enteriditis infections. In the 1980s, high rates of *Salmonella* Enteriditis infections primarily associated with shell eggs were recognized, and a variety of prevention and control measures were put in place to combat these rising rates (*16*). The Northeast was particularly affected by this outbreak; New York state reported the highest number of outbreaks during 1985–1999 (*16*). Prevention measures appeared to have the greatest success in reducing rates in the Northeast compared with others (*16*). Furthermore, 2 notable changes in NYC during this time period led to improved surveillance, which typically results in better detection and reporting. In 2006, the NYC Board of Health mandated electronic laboratory reporting of notifiable diseases (*17*); in 2009, NYC became a Foodborne Diseases Centers for Outbreak Response Enhancement (FoodCORE) center with the goal of improving surveillance for *Salmonella* infections (*18*). To the extent that these efforts may have improved surveillance in NYC, they may also have been expected to increase case detection in NYC versus NYS.

The implementation of letter grading in 2010 marks the beginning of the current declining trend in *Salmonella* infections. By improving sanitation conditions in NYC food service establishments, the letter grade program can be expected to benefit other areas as well. In this study, *Salmonella* infection were reported by county of residence, but NYC sees its population change daily due to commuting and tourism. Manhattan, one of the 5 NYC boroughs, sees its population nearly double during the workday. Of its commuters, 36% (≈550,000 persons) travel from outside the other 4 boroughs (*19*). Additionally, NYC is a popular place for tourists; >60 million persons visited in 2016 (*20*). Our study showed that *Salmonella* infections in NYS declined after the letter grade program was implemented. This finding may be in part because improved sanitary conditions in NYC restaurants after the implementation of letter grades reduced risk for *Salmonella* exposure among NYS residents who commuted to or visited NYC.

This study had several limitations. First, this was a quasi-experimental, ecologic study that represents an association and not a causal relationship. Second, the NYC restaurant letter grade program involved multiple changes to sanitation enforcement in addition to letter grade posting; changes included inspection frequency, greater risk for fines, improvements to online resources, and additional training opportunities (*21*). As a result, we could not determine which factors contributed the most to the reduction in *Salmonella* infections. Furthermore, we were not able to assess whether *Salmonella-*infected persons had a known exposure to restaurants in the period before illness.

This study supports findings from an earlier NYC study (*21*) and previous studies of Los Angeles County that showed a decline in foodborne illness hospitalizations. Hospitalizations represent a subset of foodborne illnesses that may be caused by a variety of agents, such as *Campylobacter*, another leading cause of foodborne illness in the United States (*1*). However, *Campylobacter* rarely causes outbreaks in restaurant settings because its biology limits transmission to inadequate cooking of contaminated poultry or meats or cross-contamination from raw to ready-to-eat foods (*22*). As a result, improvements in restaurant sanitary conditions are unlikely to affect *Campylobacter* transmission in restaurants.

In contrast, the selection of *Salmonella* infections is a strength of this study because the biology of *Salmonella* makes it uniquely suited for transmission in food service establishments. In restaurants, *Salmonella* can cause illness from contaminated raw ingredients, through cross-contamination, or from infected food workers. Improper cooling of inadequately cooked foods, or failure to maintain cold or hot holding temperatures can amplify contamination. Thus, *Salmonella* transmission serves as a good indicator of overall restaurant food safety practices.

Despite limitations, NYC’s experience provides a useful case study of the beneficial effect of letter grading programs. Although the relationship between restaurant inspections and risk for foodborne illness is not well understood (*23*) and inspections represent a snapshot in time that may not represent the overall sanitary conditions in restaurants, factors related to food handling and preparation practices and food worker health and hygiene are frequent contributors to outbreaks in restaurants (*24*). These factors probably contribute to transmission of sporadic infections in restaurants, which are much more common; outbreak cases represent <10% of all *Salmonella* infections (*4**,**6*). The NYC restaurant letter grade program has been shown to be associated with sustained improvements in sanitary conditions in restaurants, including several factors associated with outbreaks (*9*). Furthermore, that study showed that after 18 months, 81% of adults in NYC had seen letter grade placards, and 88% of those persons considered the letter grades in their dining decisions (*9*). This finding suggests that consumer behavior helped support the program goal of driving improvements in sanitary conditions. Although future studies are needed to parse which restaurant inspection results may contribute most to declines in *Salmonella* infections, our findings support the hypothesis that the successful implementation of a letter grade program was associated with a reduction of *Salmonella* transmission in restaurants in NYC.

In conclusion, in the United States, considerable resources have been invested to prevent contamination of the food supply before the point of service. However, *Salmonella* infections remain unchanged at the national level. Previous studies have shown improvements in sanitary conditions after the implementation of the NYC restaurant letter grade program, and our study suggests a beneficial effect on the incidence of foodborne illnesses. Implementing a letter grade program is a feasible and relatively inexpensive tool to reduce *Salmonella* infections that warrants consideration for broader use. Other jurisdictions should consider adopting a letter grade program and decide on the form and location of the placard, frequency of inspections, and approaches to engage restaurant-industry and community support to ensure program success.
